# To Prevent Blindness

**Published:** 1909-08

**Authors:** 


To Prevent Blindness.—Ophthalmia neonatorum, as everybody knows, causes the greater number of cases of blindness. It is the one cause of congenital blindness that we always think of. It is preventable. It ought to be criminal for any obstetrician or midwife to allow a case to develop under his or her care, unless he can show that he made strenuous efforts to cope with the disease. If such effort has been made, loss of vision is the exception. Some of our Legislatures will this winter have bills presented favoring better legislation on this matter. There should be some way of attaching the blame. Every case of ophthalmia should be promptly reported along with detailed measures adopted for its alleviation. Energetic prophylaxis should be required in this matter when cases occur in the slums and among the ignorant. There should be a dissemination of knowledge on this subject among those who are about to become mothers if there should be the least suspicion of venereal disease. Let us have laws by which we may know who are wilfully negligent in the accouchement chamber.—*Medical Summary.*

